# Renal Venous Anatomy in 192 Human Kidneys: Morphometry, Hilar Topography, and Anatomical Variants

**DOI:** 10.3390/jimaging12100513

**Published:** 2026-10-15

**Authors:** Stere Popescu, Florin Gabriel Panculescu, Florin Daniel Enache, Lucian Serbanescu, Mihai Victor Lupascu, Teodor Stefan Nitu, Irina Nitu, Maria Sabina Neacsu, Ioana Livia Suliman, Ana Maria Grigorescu, Iulian Manac, Alexandru Vicentiu Valcu, Andreea Cristina Costea

**Affiliations:** 1 Faculty of Medicine and Pharmacy, Ovidius University, 900470 Constanța, Romania; stelu_popescu@yahoo.com (S.P.); gabriel.panculescu@yahoo.ro (F.G.P.); dr_lupa@yahoo.com (M.V.L.); nituteodorstefan@gmail.com (T.S.N.); irris_24@yahoo.com (I.N.); neacsu.m.sabina@gmail.com (M.S.N.); panculescu_i@yahoo.com (I.L.S.); anna_grigorescu@yahoo.com (A.M.G.); iulian.manac@gmail.com (I.M.); valcu_aleex@yahoo.com (A.V.V.); acostea100@yahoo.com (A.C.C.); 2 General Surgery Department, “Sfântul Apostol Andrei” Emergency County Clinical Hospital, 900591 Constanța, Romania; 3 Diabetes Department, “Sfântul Apostol Andrei” Emergency County Clinical Hospital, 900591 Constanța, Romania; 4 Pediatric Surgery Department, “Sfântul Apostol Andrei” Emergency County Clinical Hospital, 900591 Constanța, Romania; 5 Gynecology Department, “Sfântul Apostol Andrei” Emergency County Clinical Hospital, 900591 Constanța, Romania; 6 Otolaryngology Department, “Sfântul Apostol Andrei” Emergency County Clinical Hospital, 900591 Constanța, Romania; 7 Nephrology Department, “Sfântul Apostol Andrei” Emergency County Clinical Hospital, 900591 Constanța, Romania; 8 Orthopedic Department, “Sfântul Apostol Andrei” Emergency County Clinical Hospital, 900591 Constanța, Romania

**Keywords:** renal vein, posterior renal vein, anatomical variation, corrosion cast, CT angiography, retroaortic left renal vein, circumaortic renal vein, nephrectomy, living kidney donor

## Abstract

Renal venous anatomy is relevant to nephrectomy, living-donor evaluation, and retroperitoneal surgery. Classical descriptions often present a single renal vein on each side, whereas published series show that additional veins, retroaortic and circumaortic courses, and variable hilar tributaries are common. This study describes formation patterns, morphometry, and variants in four separate, non-overlapping methodological series of surgical specimens and imaging examinations, with particular attention to the termination of the posterior primary venous branch, the types of venous anastomosis, and the relation of the vein to the artery at the hilum. We examined 192 adult human kidneys (93 right, 99 left) in four separate methodological series, with no kidney examined by more than one technique: anatomical dissection of nephrectomy specimens (*n* = 64), methacrylate injection and corrosion casting of nephrectomy specimens (*n* = 93), contrast-enhanced CT angiography (*n* = 23), and barium injection with radiography of nephrectomy specimens (*n* = 12). The kidney was the unit of analysis. Because some right and left kidneys may have originated from the same individual and the pairing structure could not be reconstructed, right- and left-sided findings are reported descriptively, without inferential side comparisons. Each feature was analysed only in the kidneys in which it could be assessed. The four techniques differ in their ability to detect small tributaries and anastomoses, and frequencies pooled across methods are descriptive. Among 122 kidneys assessed for hilar formation, the renal vein was formed of two primary trunks in 76/122 (62.3%; 95% CI 53.4–70.4), three trunks in 34/122 (27.9%), and four trunks in 12/122 (9.8%). The confluence was assessed in 110 kidneys and was intrasinusal in 40/110 (36.4%), juxtahilar in 34/110 (30.9%), and extrarenal in 36/110 (32.7%). A posterior primary venous branch was present in 56/122 (45.9%): 19/50 right (38.0%) and 37/72 left (51.4%); it joined the already formed main trunk in 32/56 and took part in the confluence in 14/56. In 90 kidneys the main vein lay anterior to the artery in 42/90 (46.7%). In the descriptive morphometric summary of 78 veins, mean length was 6.9 cm on the left (range 4.8–12.5) and 2.9 cm on the right (range 1.5–5.5). At least one venous anastomosis was recorded in 77/192 kidneys (40.1%; 95% CI 33.4–47.2; pooled across methods): 45/93 right (48.4%; 95% CI 38.5–58.4) and 32/99 left (32.3%; 95% CI 23.9–42.0). Multiple renal veins were identified in 16/116 assessable kidneys (13.8%), including 14/52 right (26.9%) and 2/64 left (3.1%). A retroaortic left renal vein was seen in 2/42 assessed left veins (4.8%), and a complete circumaortic ring in one left specimen. The renal vein was often formed of more than one primary trunk, a posterior branch was present in about half of the kidneys assessed, and multiple veins predominated on the right, in line with previous surgical and imaging series. The more specific contributions of this material are the posterior-branch termination map, the classification of anastomosis types, and the vein–artery counts at the hilum. The study is anatomical and descriptive. It does not measure surgical outcomes and does not show that additional venous imaging is required in every patient.

## 1. Introduction

The renal veins drain the kidney, the perinephric tissues, and, on the left, major adrenal, gonadal, and lumbar tributaries [1]. Standard textbook accounts still describe a single renal vein on each side, with limited attention to hilar formation, posterior tributaries, or course variants [1]. That description is incomplete. Additional renal veins, circumaortic and retroaortic left renal veins, and variable primary tributaries have been documented in cadaveric, surgical, and imaging series for decades [2,3,4,5].

Hostiuc and colleagues, in a meta-analysis of 105 studies, estimated the prevalence of multiple renal veins at 16.7% (14.3–19.2%), about eight times higher on the right (16.6%) than on the left (2.1%); retroaortic left renal vein at 3.0% (2.4–3.6%); and circumaortic left renal vein at 3.5% (2.8–4.4%) [5]. Satyapal reported a single additional renal vein in 26% of right kidneys and 2.6% of left kidneys [6] and proposed a practical classification based on primary tributaries and additional veins [7]. In 342 living-donor nephrectomies, Baptista-Silva and colleagues found the left renal vein to be single in every left kidney, whereas the right vein was double or triple in more than one third of right-sided grafts [8]. These figures already show that venous variability is the expected anatomy, not an exception.

Rarer configurations also occur and can crowd the hilum. Nayak and colleagues described five independent right renal veins entering the inferior vena cava through separate openings [9], multiple vascular variations around the left kidney including a retropelvic venous tributary [10], and, on the arterial side, eight prehilar branches of the right renal artery producing a congested hilum [11,12,13]. Such case reports do not change prevalence estimates, but they show why a “single vein, single artery” mental model is unsafe in the individual patient.

Unrecognised venous variants can complicate donor nephrectomy, tumour thrombectomy, para-aortic lymphadenectomy, and aortic exposure [14,15,16]. Accurate identification may become particularly challenging in catastrophic aviation disasters, where extensive traumatic destruction and fragmentation can limit the availability of conventional identifying features; systematic forensic approaches are therefore essential [12,13]. A retroaortic left renal vein may be asymptomatic. Compression between the aorta and the vertebral column is the anatomical substrate of the posterior nutcracker phenomenon; nutcracker syndrome is reserved for the symptomatic patient and is not a synonym for the variant itself [12,17].

Most published series use one method. Dissection, corrosion casting, radiography, and CT angiography do not have the same detection threshold for small tributaries or anastomoses [4,5,18]. Combining them can widen the anatomical description, but the methods should be kept apart wherever the records allow, and the denominator should be stated for every feature.

The present study describes 192 adult kidneys that originated from four separate methodological series; each kidney was examined by one method only, and feature-specific denominators are reported where available. For some outcomes, in particular venous anastomoses, method-specific numerators were not retained, so these results are necessarily pooled across methods and are presented as descriptive frequencies. The aims were to record (i) the number and site of primary venous trunks, (ii) the presence and termination of a posterior primary branch, (iii) the topographical relation of the vein to the artery, (iv) length and diameter in the subset with morphometric summaries, (v) visible venous anastomoses, and (vi) selected course and number variants, and to place these findings against published prevalence estimates rather than against a simplified textbook drawing. Several of these features, namely the predominance of two primary trunks, the longer left vein, and the right-sided predominance of multiple veins, are already well established, and the study does not claim to describe them for the first time. Its more specific contributions are threefold: a termination map of the posterior primary venous branch with side-specific counts, a classification of visible venous anastomoses by type, and counts of the antero-posterior relation of the renal vein to the renal artery at the hilum.

## 2. Materials and Methods

This anatomical and imaging study included 192 adult human kidneys (93 right, 99 left), which originated from four separate, non-overlapping methodological series; no kidney was examined by more than one technique. Anatomical dissection (*n* = 64) and methacrylate corrosion casting (*n* = 93) were performed on nephrectomy specimens submitted to the General Surgery department, “Sfântul Apostol Andrei” Emergency County Clinical Hospital, Constanța. Barium injection and radiography (*n* = 12) were performed on additional nephrectomy specimens that were not used for dissection or corrosion casting. Contrast-enhanced CT angiograms (*n* = 23) were reviewed retrospectively from the Medimar Imaging Center of the Constanța Clinical Emergency Hospital and the Euromedic Diagnostic Imaging Center, Constanța.

Adult kidneys in which the renal vein and its hilar tributaries could be displayed were eligible. The surgical indication for each nephrectomy and the underlying renal pathology were not retained in the analytical dataset, and patient-level demographic variables such as age and sex were likewise not retained. A kidney was excluded from the analysis of a given feature when that feature could not be visualised completely, and it remained available for the analysis of other features. Because the indication for nephrectomy was not recorded, an influence of the underlying disease on local venous anatomy cannot be excluded; this is addressed in Section 4.9.

The kidney was the unit of analysis. The number of unique individuals and the number of right–left pairs originating from the same individual could not be reconstructed from the retained records, although at least one such pair was documented (Section 3.6). Right and left kidneys therefore cannot be assumed to be independent observations, and for this reason no inferential side comparisons were performed (Section 2.6). Table 1 shows an overview of investigation methods used for the case studies.

### 2.1. Anatomical Dissection

Anatomical dissection was carried out on 64 kidneys (Table 2), prepared as fresh or formalin-fixed specimens according to standard laboratory protocol. In a subset of cases the kidney was removed with the perirenal fat, a segment of abdominal aorta, and a segment of inferior vena cava still in continuity. The aim of dissection was to display the renal vein or veins, their primary tributaries, and their relation to the renal artery and the collecting system. Length and external diameter were measured with calibrated digital calipers (resolution 0.1 mm) at the landmarks defined in Section 2.5.

### 2.2. Plastic Injection and Corrosion Casting

Plastic injection and corrosion were applied to 93 kidneys (Table 3). The artery and vein were cannulated separately and injected with Technovit 7143, a self-polymerising methacrylate resin. Injection was continued until the surface tributaries filled, under gentle, manually controlled pressure that was not instrumentally measured. After polymerisation for 24–48 h, the soft tissue was digested in heated potassium hydroxide (30% KOH, 60 °C). Sodium hydroxide was not used. Casts were rinsed, dried, photographed under a fixed camera distance, and measured on calibrated photographs.

### 2.3. Computed Tomography and CT Angiography

Twenty-three contrast-enhanced CT angiography examinations, obtained from the Medimar Imaging Center at the Constanța Clinical Emergency Hospital and the Euromedic Diagnostic Imaging Center in Constanța, were reviewed retrospectively. All examinations were acquired on a GE LightSpeed 16-slice scanner (GE Healthcare, Chicago, IL, USA) after intravenous injection of non-ionic iodinated contrast (iohexol, 300 mg I/mL, 1.5 mL/kg). Arterial and venous phases were obtained with bolus tracking, using a fixed institutional delay protocol optimised for venous-phase opacification. Images were reconstructed in axial, coronal, and sagittal planes and reviewed as maximum-intensity-projection and volume-rendered series. Because the examinations were retrieved retrospectively from routine clinical practice, the individual acquisition parameters (tube voltage, tube current–time product, slice thickness, reconstruction interval, field of view, and in-plane resolution) were not retained in the study dataset and are not reported. A renal vein or tributary was recorded only when it could be followed on contiguous slices from the hilum to its termination. Given the spatial resolution of a 16-slice system, small tributaries and fine communicating channels are expected to be under-detected on CT relative to corrosion casting, and CT findings were interpreted accordingly.

### 2.4. Barium Injection and Radiography

In an additional 12 nephrectomy specimens, barium sulfate suspension (100% *w*/*v*) was injected through the renal vein under gentle manual pressure, which was not instrumentally recorded, followed by anteroposterior and lateral radiographic exposures at a standardised, fixed film-focus distance. These radiographs were used for qualitative mapping of venous trunks and anastomoses. They were not pooled with caliper or CT measurements.

### 2.5. Measurement Protocol and Reproducibility

All measurements were made by one observer experienced in renal vascular anatomy. Each length and diameter was measured twice, and the mean of the two readings was entered in the analytical dataset; the individual duplicate readings were not retained. Intraobserver reliability, which would normally be quantified from such paired readings with the intraclass correlation coefficient (ICC), the technical error of measurement (TEM), the relative TEM, and the coefficient of reliability (R), therefore could not be calculated retrospectively. Because only one observer took part, interobserver agreement could not be assessed either. Both points are limitations of the morphometric component and they are mentioned in Section 4.9.

Definitions used throughout the paper:

- Caliber means external diameter of the vein, in centimetres, at a stated landmark. It is not a flow measurement.
- Origin of the renal vein is the point at which the primary trunks have united into a single extra-hilar vein, or, when several veins remain separate, the hilar exit of each vein.
- Termination is the junction of that vein with the inferior vena cava.
- Length is the shortest distance along the vein from origin to termination.
- A primary trunk is a first-order tributary that leaves the sinus or hilum and joins another first-order tributary to form the renal vein, or that reaches the inferior vena cava on its own.
- Formation site is intrasinusal if the definitive vein is already formed inside the sinus; juxtahilar if the confluence lies at the hilar margin; and extrarenal if the confluence lies medial to the hilum.
- A posterior primary venous branch is a first-order tributary that leaves the kidney behind the renal pelvis and either joins the main renal vein or reaches the inferior vena cava separately [19].
- A venous anastomosis is a communicating channel joining two renal venous trunks, two primary tributaries, or a renal vein and another named vein, other than the ordinary end-to-side entry of a single adrenal, gonadal, or lumbar tributary into one trunk. This is an anatomical definition. Patency under physiological flow was not tested.
- Multiple renal veins means two or more veins leaving the kidney and entering the inferior vena cava through separate ostia [2,5].
- Retroaortic left renal vein is a left renal vein that crosses behind the abdominal aorta to reach the inferior vena cava [5,13].
- Circumaortic venous ring comprises preaortic and retroaortic left venous limbs that together encircle the aorta [5,13].

Terminology followed *Terminologia Anatomica* [18]. Kidneys in which a given feature could not be seen completely were omitted from that analysis only.

### 2.6. Statistical Analysis

Each anatomical parameter was analysed only in the kidneys in which it was assessable. Descriptive statistics were obtained in SPSS version 30.0 (IBM Corp., Armonk, NY, USA) [20]. Categorical variables are given as n/N and percentages, with 95% confidence intervals calculated by the Wilson score method. Continuous variables are given as mean and observed range. Because some right and left kidneys may have originated from the same individual and the pairing structure could not be reconstructed, the assumption of independent observations required by the chi-square and Fisher’s exact tests could not be verified. No inferential side comparisons were therefore performed, and right- and left-sided findings are reported descriptively as n/N, percentages, and 95% confidence intervals. Length and diameter are likewise reported descriptively, because only summary values (mean and range per side) were retained and the measurements pooled methods that are not metrically interchangeable (Section 3.4). The four investigative methods were not compared statistically, because they were applied to different kidneys and do not have the same detection threshold. Frequencies pooled across methods are presented as descriptive estimates, not as method-independent prevalence.

## 3. Results

### 3.1. Formation Patterns of the Renal Vein

The number of primary trunks was assessed in 122 kidneys (50 right, 72 left; Table 4). The most common pattern was formation from two trunks, in 76/122 (62.3%; 95% CI 53.4–70.4): 28/50 right (56.0%; 95% CI 42.3–68.8) and 48/72 left (66.7%; 95% CI 55.2–76.5). Three trunks were present in 34/122 (27.9%) and four trunks in 12/122 (9.8%).

The site of confluence was assessed in 110 kidneys. The vein formed inside the sinus in 40/110 (36.4%), at the hilar margin in 34/110 (30.9%), and extrarenally in 36/110 (32.7%). Extrarenal formation was more frequent on the left side (35.9% of left kidneys assessed for this feature) than on the right, and the extrarenal confluence was often located halfway between the kidney and the aorta or closer to the aortic wall.

### 3.2. Posterior Primary Venous Branch

**Figure 1:**
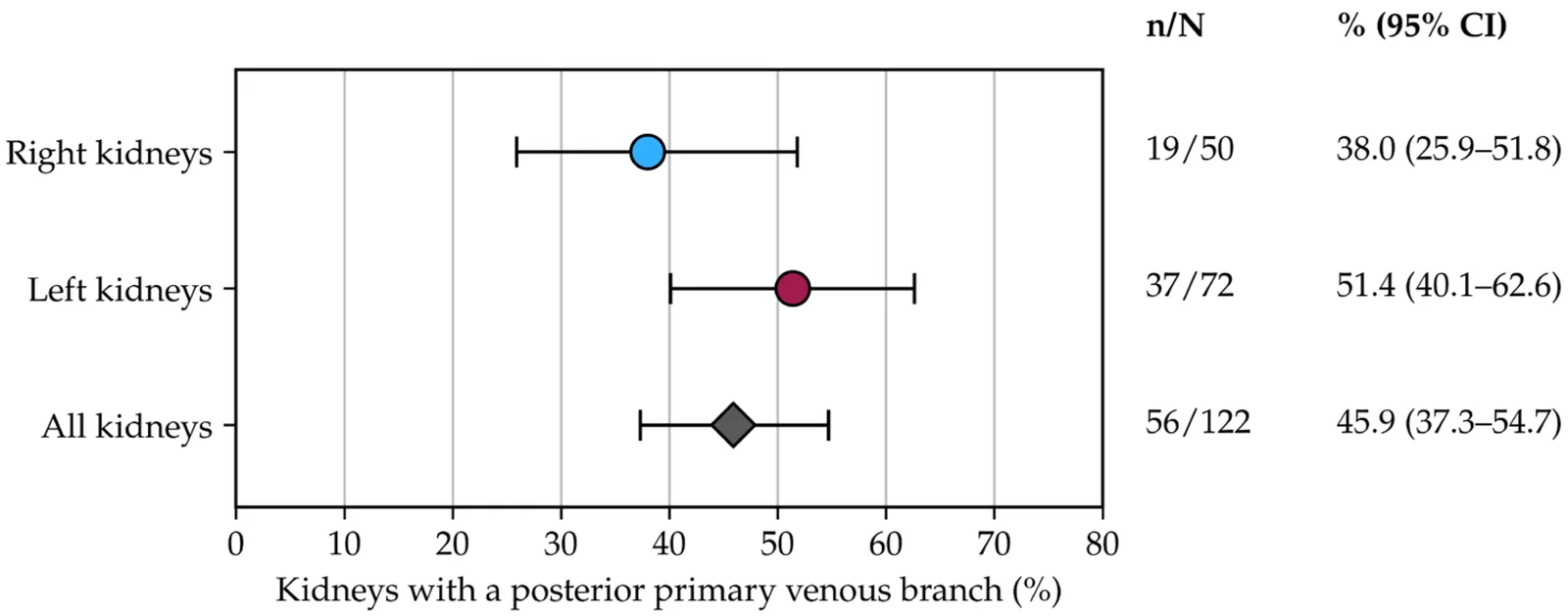
Prevalence of a posterior primary venous branch in the 122 kidneys assessed for this feature (50 right, 72 left). Circles show the percentage of right and left kidneys with the branch and the diamond shows the percentage for all kidneys; horizontal bars are 95% confidence intervals calculated by the Wilson score method. The numerator and denominator of each estimate are listed on the right.

The presence and configuration of a posterior primary venous branch (ramus posterior) were evaluated in 122 kidneys (Table 5, Figure 1). A posterior branch was present in 56/122 (45.9%; 95% CI 37.3–54.7): 19/50 right (38.0%; 95% CI 25.9–51.8) and 37/72 left (51.4%; 95% CI 40.1–62.6).

Among the 56 kidneys with a posterior branch, the branch joined the already formed main trunk in 32/56 (57.1%), took part in the confluence itself in 14/56 (25.0%), joined an antero-superior trunk in 6/56 (10.7%), an antero-inferior trunk in 2/56 (3.6%), a mesorenal anterior trunk in 1/56 (1.8%), and reached the inferior vena cava as a separate vein in 1/56 (1.8%). Side-specific counts are given in Table 5. An arciform course was recorded in 22/56 (39.3%).

### 3.3. Anatomical Relationships Between the Renal Vein and Artery

Vein–artery topography was assessed in 90 well-preserved kidneys, 45 on each side (Table 6). The renal vein lay anterior to the corresponding artery in 42/90 (46.7%; 95% CI 36.7–56.9): 24/45 right (53.3%; 95% CI 39.1–67.1) and 18/45 left (40.0%; 95% CI 27.0–54.5). A posterior position of the vein relative to the artery was seen in 11/90 (12.2%; 95% CI 7.0–20.6), all on the left (11/45 left kidneys, 24.4%). In the remaining 37/90 kidneys (41.1%; 21/45 right and 16/45 left) the two vessels overlapped only in part and did not lie in a single coronal plane.

### 3.4. Renal Vein Morphometry

Descriptive morphometric summaries were available for 78 renal veins (36 right, 42 left; Table 7). These values pool measurements obtained by dissection, on corrosion casts, and on CT. The methods are not metrically interchangeable, because fixation, injection pressure, and voxel size all affect vessel dimensions, and the method of origin of each morphometric record was not retained. Only the mean and the observed range per side were retained; individual measurements and standard deviations are not available. Table 7 is therefore a descriptive summary, and no formal side comparison is reported.

Mean length was 6.9 cm on the left (range 4.8–12.5) and 2.9 cm on the right (range 1.5–5.5). Mean diameter was larger at termination than at origin on both sides (left 0.97 to 1.22 cm; right 0.82 to 1.05 cm). The direction of the length difference is the classical one [1,8].

### 3.5. Venous Anastomoses

At least one communicating venous channel, as defined in Section 2.5, was recorded in 77/192 kidneys (40.1%; 95% CI 33.4–47.2): 45/93 right (48.4%; 95% CI 38.5–58.4) and 32/99 left (32.3%; 95% CI 23.9–42.0) (Table 8). This is a pooled descriptive frequency. Its denominator assumes that the feature could be judged in every kidney, and the four methods differ in their ability to show small communications: corrosion casts are expected to reveal them more readily than 16-slice CT or barium radiographs. Method-specific numerators were not retained, so the pooled value should not be read as a method-independent prevalence. The side-specific values are given descriptively; no inferential side comparison was made, for the reason given in Section 2.6.

Among the 77 kidneys with at least one anastomosis, the communication was extrarenal and intertruncular in 31/77 (40.3%), juxtahilar anterior in 19/77 (24.7%), intrasinusal in 14/77 (18.2%), related to a retroaortic course in 8/77 (10.4%), and lobar or segmental in 5/77 (6.5%). Side-specific counts for the individual anastomosis types were not tabulated, so the distribution of types is reported for both sides combined.

### 3.6. Variants and Anomalies of the Renal Vein

Course and number variants were recorded only in the kidneys in which the full path of the vein to the inferior vena cava could be seen (Table 9).

A retroaortic left renal vein was identified in 2 of 42 assessed left veins (4.8%; 95% CI 1.3–15.8). In both specimens the vein crossed behind the aorta and entered the posterolateral wall of the inferior vena cava. One of these kidneys was associated with a duplicated right renal vein in the same person, and the other with an incomplete venous ring. Neither specimen had clinical data that would allow a diagnosis of nutcracker syndrome. The finding is therefore reported as an anatomical variant [12,13].

A complete circumaortic ring, with preaortic and retroaortic limbs joining both ends of the left hilum and receiving adrenal and lumbar tributaries, was identified in one specimen. Because course-variant screening for this specific configuration was not tabulated against a separate denominator, the finding is reported descriptively as a single case rather than as a prevalence estimate.

Multiple renal veins, defined as separate caval ostia, were identified in 16 of 116 assessable kidneys (13.8%; 95% CI 8.7–21.2): 14 of 52 right kidneys (26.9%; 95% CI 16.8–40.3) and 2 of 64 left kidneys (3.1%; 95% CI 0.9–10.7). Three of the right-sided cases had three veins, one of which was an inferior polar vein.

**Figure 2:**
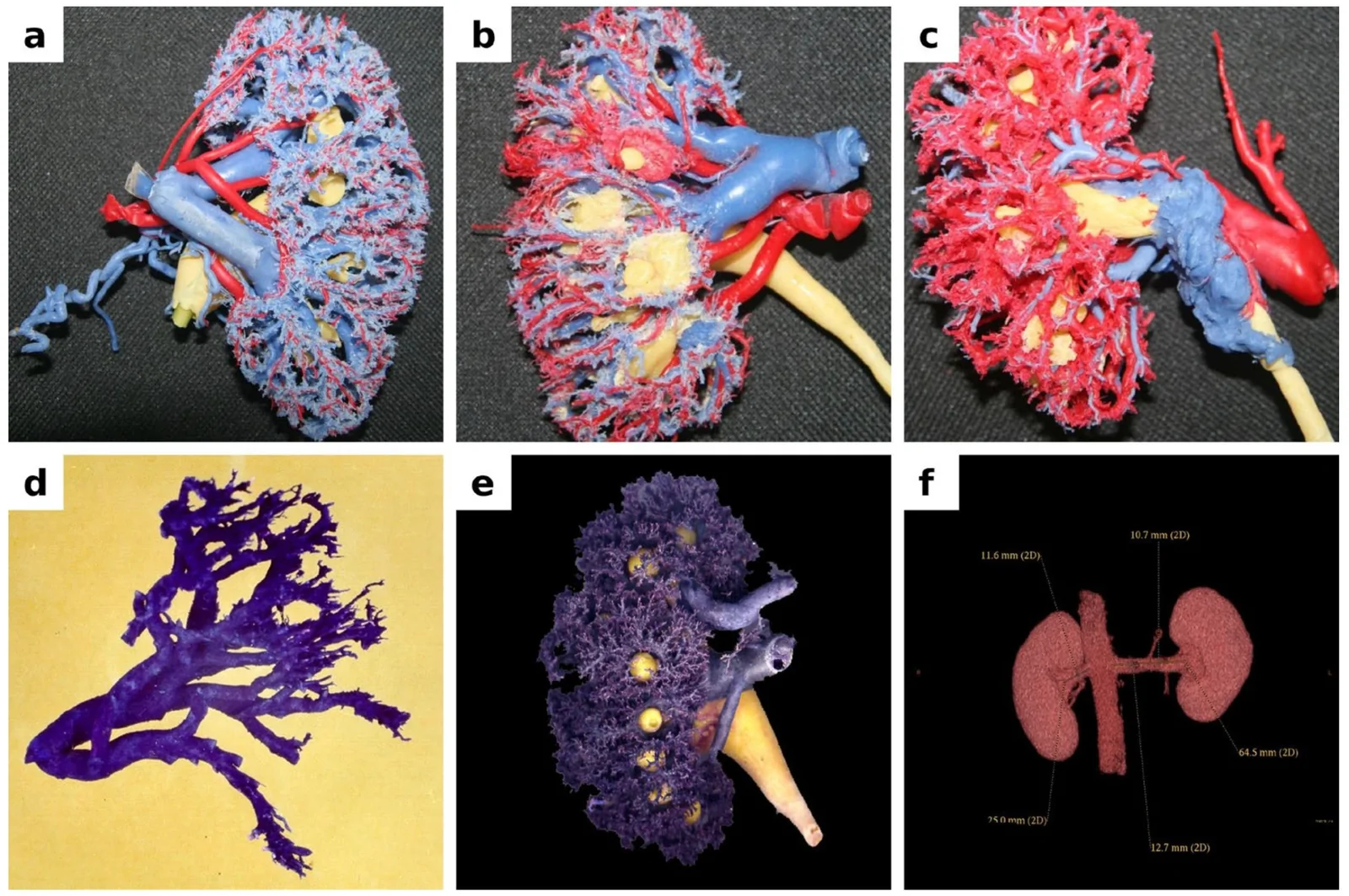
Representative images from the present series. Panels (**a**–**e**) are methacrylate corrosion casts of nephrectomy specimens (veins blue, arteries red, collecting system yellow in the multicolour casts); panel (**f**) is a contrast-enhanced CT angiogram. (**a**) Left kidney, anterior view: the renal vein trunk and its intrarenal branches lie anterior to their arterial counterparts. (**b**) Right kidney, anterior view: the renal vein trunk lies above the artery; at the hilum the primary venous branches lie anterior to the arterial branches in the upper half of the kidney and posterior to them in the lower half. (**c**) Left kidney, posterior view: posterior primary venous branch forming an arcade. (**d**) Venous cast, posterior view: renal vein formed of two venous trunks, with the posterior primary branch ending in the antero-inferior trunk. (**e**) Right kidney, posterior view: two right renal veins; the lower, larger vein receives an inferior polar branch near the hilum, and a curved extrarenal anastomosis, concave laterally, connects the two veins. (**f**) CT angiography, volume-rendered image, anterior view, with measurements: left renal vein 64.5 mm long, 10.7 mm in calibre at its origin and 12.7 mm at its termination; right renal vein 25.0 mm long and 11.6 mm in calibre. Patient identifiers have been removed from the image.

Figure 2 shows some representative images from the present series.

## 4. Discussion

This study describes renal venous anatomy in 192 kidneys examined by one of four methods. The methods were not applied to the same organs and do not have the same sensitivity for small tributaries. The frequencies are therefore those of four separate methodological series that happen to have been collected in the same institution, not the output of a formal method-comparison study. Read that way, the material is still useful: it records how often the vein is formed of more than two trunks, how a posterior branch ends, how the vein sits relative to the artery, and how often a second caval ostium is present.

### 4.1. Number and Site of Primary Trunks

Formation from two primary trunks in 76/122 kidneys (62.3%) is compatible with Satyapal’s Type I pattern, which was also the most common configuration in his resin-cast and plastination series [7]. Three or four trunks in more than one third of the present kidneys is not a new discovery. It is the reason Satyapal proposed a classification that already includes more than two primary tributaries [7,21]. What the present series adds is a side-stratified count from a mixed Romanian surgical and imaging sample, and a reminder that hilar dissection should expect more than one anterior tributary.

Satyapal’s full typology was not applied, because additional veins and primary-tributary number were not coded on the same record sheet for every kidney. Applying that typology prospectively would allow a direct comparison with his series.

### 4.2. Posterior Primary Branch

A posterior primary branch was present in 56/122 kidneys (45.9%): 19/50 right (38.0%) and 37/72 left (51.4%), with overlapping 95% confidence intervals (Figure 1). No inferential side comparison was made, and the present data do not establish a left-sided predominance. Okamoto, using a related definition, found a vein behind the pelvis at the hilum in about 80% of kidneys and described typical and non-typical drainage of that vein into the cava or into the anterior renal vein [19]. Against that benchmark the present prevalence is not high. It is lower, and the difference is more likely to reflect definition and method than a newly recognised left-sided structure.

The practical point is the termination pattern, not the headline percentage. In 14/56 positive cases the posterior branch took part in the confluence itself, and in 22/56 it had an arciform course. Those configurations can sit behind the pelvis during posterior mobilisation and during partial nephrectomy [22,23]. They should be looked for; they should not be described as previously unreported.

### 4.3. Vein–Artery Relationship at the Hilum

An anterior vein in 42/90 kidneys (46.7%) is consistent with the usual textbook order, but it was not the only pattern. A posterior vein in 11/90, all on the left, partial overlap of the two vessels in 37/90 (Table 6), and a superior vein in 26/90 show that the hilum is not a fixed stack of vein–artery–pelvis. That observation matters for laparoscopic and robotic hilar dissection, where the first structure seen is not reliably the vein [22,23]. The present study did not record operative times or injuries, so it supports anticipation, not a change in surgical approach.

### 4.4. Morphometry

A longer left renal vein (mean 6.9 cm versus 2.9 cm) is the classical reason for preferring the left kidney in living donation [1,8]. Hostiuc and colleagues quoted mean lengths of about 8.4 cm on the left and 3.2 cm on the right [5]. The present means lie in the same direction and are slightly lower, which would be expected if part of the measurements were taken on fixed tissue or on casts. Because the 78 values pool methods that are not metrically interchangeable, and because only means and ranges were retained, these figures are descriptive only: no *p* value is reported, and the left-to-right ratio is not proposed as a morphometric reference value.

Baptista-Silva and colleagues, in their series of 342 living-donor nephrectomies, found the left renal vein to be single in essentially all left-sided specimens, with constant adrenal and gonadal tributaries and a renolumbar vein in a substantial proportion of cases [8]. Shreevastava and colleagues similarly described protean drainage patterns of the left renal vein in a surgical series, reinforcing that tributary anatomy on the left side is more variable than a single-vessel textbook description suggests [24]. That surgical evidence explains why left-sided complexity in the present material is mainly tributary anatomy, not a second caval ostium.

### 4.5. Anastomoses

Communications were recorded in 77/192 kidneys (40.1%): 45/93 right (48.4%) and 32/99 left (32.3%). These side-specific values are descriptive, and no inferential side comparison was made. This pooled figure combines methods with different detection thresholds and is best read as a descriptive frequency in this material rather than as a prevalence. The definition used here is morphological. Selberherr and colleagues have shown, in a different setting, that named left-sided collaterals can preserve renal function after planned division of the left renal vein close to the cava [25]. That is functional evidence from aortic surgery. It does not follow that every cast communication in the present series would do the same after ligation or tumour thrombus. We therefore do not describe these channels as a “dynamic network” and we do not claim that they are previously unreported realities accessible only by corrosion casting.

Hoff and colleagues noted that supernumerary transplant veins can often be ligated because venous territories overlap, but that the decision is still individual and may require on-table assessment [26]. That is the correct clinical register for the present anatomical findings.

### 4.6. Number and Course Variants

Multiple renal veins in 16/116 kidneys (13.8%), including 26.9% of right and 3.1% of left assessable kidneys, sit inside the ranges already published by Satyapal (26% right, 2.6% left) and by Hostiuc (16.6% right, 2.1% left) [5,6]. The present series confirms the laterality. It does not extend it.

A retroaortic left renal vein in 2/42 assessed left veins (4.8%) is compatible with the pooled 3.0% estimate, given the wide confidence interval of a small subset [5,27]. A single circumaortic ring is a recognised variant, not a new one [4,5]. Damen et al. have recently shown how much finer the retroaortic typology can be on CT angiography [27]. The present study was not designed for that typology.

Rarer crowding of the hilum, such as five separate right renal veins [9] or a left retropelvic tributary with an IVC communication [10], was not seen in this material. Those cases are cited so that the Discussion is not limited to the variants we happened to count.

A retroaortic left renal vein is not nutcracker syndrome. Posterior nutcracker phenomenon refers to compression of a retroaortic vein between the aorta and the spine. The syndrome requires the corresponding clinical picture [12]. Neither was ascertained here.

The pelvicalyceal system was displayed together with the renal vein in 50 dissected kidneys and 72 injected kidneys (Table 2 and Table 3), mainly to define the posterior primary branch relative to the renal pelvis and the antero-posterior order of the hilar structures. Pelvi-ureteric variants, such as a bifid or extrarenal pelvis or a duplicated ureter, were not coded systematically, so their frequency and any association with renal venous variants cannot be reported from this material. Co-occurrence of vascular and pelvic anomalies has been described [12], and a retropelvic venous tributary is one configuration in which the two systems are directly related [10]. A prospective series that codes venous and pelvi-ureteric anatomy on the same record would be needed to test such associations.

### 4.7. Surgical Implications, Stated as Anatomy Rather than as a Protocol

Unrecognised additional veins and retroaortic limbs have caused bleeding in retroperitoneal and aortic surgery for many years [15,16]. Preoperative venous-phase CT is already standard in living-donor evaluation and is often available before complex partial nephrectomy or tumour thrombectomy [17,22]. The present anatomical series cannot show that adding dedicated CT angiography or MR venography improves outcomes in every nephrectomy, every adrenalectomy, or every retroperitoneal resection. We therefore do not recommend additional venous imaging as a universal step. We do recommend that, when venous anatomy will determine the operation, the available images be reviewed specifically for the number of caval ostia, a posterior hilar tributary, and a retroaortic or circumaortic left limb.

### 4.8. What Is and Is Not New

The findings that confirm previous work are a two-trunk predominance, a longer left vein, multiple veins mainly on the right, and occasional retroaortic or circumaortic left veins [2,3,4,5,6]. The findings that are more specific to this series are the termination map of the posterior branch with side-specific counts (Table 5), the classification of anastomosis types (Table 8), and the vein–artery counts in 90 kidneys (Table 6). The last of these can be compared with the series of Kumar and colleagues, who examined 96 renal hila, identified twelve arrangement patterns of the hilar structures, and found the classical vein–artery–pelvis order in 45.8% and an artery–vein–pelvis order in 28.1% [28]. The proportion of kidneys with an anterior vein in the present material (46.7%) is close to their figure for the classical order, and both series show that the classical arrangement is found in fewer than half of hila. These observations are worth reporting. They are not “unreported anatomical realities”, and they do not show that only a multimodal design can describe renal venous anatomy. A single well-powered dissection or CT series can do that. A multimodal collection can do it only if the denominator of each feature is stated and results pooled across methods are identified as such.

### 4.9. Limitations

Several limitations follow from the design and from the records that were retained. First, the four methods were applied to different kidneys and differ in their detection threshold: small tributaries and anastomoses are expected to be seen more readily on corrosion casts than on 16-slice CT or barium radiographs. Method-specific numerators were not retained for the pooled features, so pooled percentages, in particular the anastomosis frequency, are descriptive and are not method-adjusted prevalence estimates. Second, the kidney was the unit of analysis, but the number of unique individuals and of right–left pairs from the same individual could not be reconstructed. Because the chi-square and Fisher’s exact tests assume independent observations, no inferential side comparisons were performed, and right–left differences are reported descriptively as n/N, percentages, and 95% confidence intervals. Third, the surgical indication for nephrectomy, the underlying renal pathology, age, and sex were not retained. Selection bias cannot be excluded, disease-related changes in local venous anatomy cannot be ruled out, and generalisability to the healthy population is limited. Fourth, the CT examinations were retrospective and their detailed acquisition parameters were not retained. Fifth, the morphometric values pool fresh tissue, fixed tissue, casts, and CT, and only means and ranges per side were retained; individual measurements, standard deviations, and the method of origin of each record are not available, which limits reproducibility and precludes formal comparison. Sixth, all measurements were made by one observer. Interobserver agreement could not be assessed because no second observer took part. Intraobserver reliability could in principle have been quantified from the duplicate readings with the ICC, TEM, relative TEM, and coefficient of reliability [29], but the individual duplicate readings were not retained, so intraobserver reproducibility is not demonstrated. Seventh, pelvi-ureteric variants were not coded, so associations between collecting-system and venous anatomy could not be examined. Finally, there are no clinical follow-up data, so statements about bleeding risk, graft outcome, or the need for extra imaging are inferences from anatomy and from other authors’ series, not results of this study. Future work should use a prospective design with patient-level linkage, method-specific recording, archived individual measurements, and formal intra- and interobserver reliability analysis.

## 5. Conclusions

In 122 kidneys assessed for hilar formation, the renal vein was formed of two primary trunks in 62.3% and by three or four trunks in the remainder. A posterior primary branch was present in 45.9% of those kidneys (38.0% right, 51.4% left); it most often joined the already formed main trunk, and in one quarter of cases it took part in the confluence itself. The renal vein lay anterior to the artery in fewer than half of the 90 kidneys assessed, and a posterior vein was seen only on the left. Multiple veins were mainly right-sided (26.9% versus 3.1%), and the left vein was longer than the right in the descriptive morphometric summary. These general patterns agree with previous surgical, donor, and imaging series; the more specific contributions of this study are the posterior-branch termination map, the classification of anastomosis types, and the vein–artery counts at the hilum. Because the methods differ in detection threshold, pairing could not be reconstructed, and the morphometric data are summary values, the results are descriptive.

Surgeons and radiologists should expect more than one hilar trunk, should look for a posterior tributary, and should not treat a retroaortic left vein as a diagnosis of nutcracker syndrome. When the planned operation depends on venous anatomy, the available venous-phase images should be read with those structures in mind. This study cannot show that additional CT angiography or MR venography is necessary in every patient.

## Figures and Tables

**Table 1:** Overview of the investigative methods used and number of cases studied for each technique.

No.	Method	No. of Cases
1	Dissection	64
2	Plastic injection/corrosion casting	93
3	CT examination (MDCT angiography)	23
4	BaSO_4_ injection/radiography	12
	Total	192

**Table 2:** Cases studied by anatomical dissection, classified by the combination of structures simultaneously examined.

No.	Dissected Element	No. of Cases
1	Renal vein	7
2	Renal vein + renal artery	7
3	Renal vein + pyelocaliceal system	6
4	Renal vein + renal artery + pyelocaliceal system	44
	Total	64

**Table 3:** Cases studied by plastic injection and corrosion, classified by the combination of injected elements.

No.	Injected Element	No. of Cases
1	Renal vein	9
2	Renal vein + renal artery	8
3	Renal vein + pyelocaliceal system	6
4	Renal vein + renal artery + pyelocaliceal system	66
	Total	93

**Table 4:** Number of primary trunks forming the renal vein, by side. Values are n (%) of the kidneys assessed for this feature.

Number of Primary Trunks	Total (*n* = 122)	Right (*n* = 50)	Left (*n* = 72)
Two	76 (62.3)	28 (56.0)	48 (66.7)
Three	34 (27.9)	16 (32.0)	18 (25.0)
Four	12 (9.8)	6 (12.0)	6 (8.3)

**Table 5:** Termination patterns of the posterior primary venous branch in the 56 kidneys in which it was present. Values are n/N (%).

Termination Site of the Posterior Branch	Total (*N* = 56)	Right (*N* = 19)	Left (*N* = 37)
Into the main renal trunk (after formation)	32/56 (57.1)	11/19 (57.9)	21/37 (56.8)
As a contributing trunk to formation	14/56 (25.0)	6/19 (31.6)	8/37 (21.6)
Into the antero-superior primary branch	6/56 (10.7)	0/19 (0.0)	6/37 (16.2)
Into the antero-inferior primary branch	2/56 (3.6)	1/19 (5.3)	1/37 (2.7)
Into the mesorenal anterior branch	1/56 (1.8)	0/19 (0.0)	1/37 (2.7)
Into the IVC as a separate vein	1/56 (1.8)	1/19 (5.3)	0/37 (0.0)

IVC, inferior vena cava. Several categories contain only one or two kidneys; the corresponding percentages are imprecise and are given for completeness only.

**Table 6:** Antero-posterior relation of the main renal vein to the renal artery at the hilum in 90 kidneys. Values are n/N (%).

Position of the Vein Relative to the Artery	Total (*N* = 90)	Right (*N* = 45)	Left (*N* = 45)
Anterior	42/90 (46.7)	24/45 (53.3)	18/45 (40.0)
Posterior	11/90 (12.2)	0/45 (0.0)	11/45 (24.4)
Partial overlap	37/90 (41.1)	21/45 (46.7)	16/45 (35.6)

Partial overlap: the vein and artery overlapped only in part and did not lie in a single coronal plane.

**Table 7:** Descriptive morphometric summary of the renal veins. Values are pooled across dissection, corrosion casts, and CT and are given as mean and observed range.

Morphometric Parameter	Left Renal Vein (*n* = 42)	Right Renal Vein (*n* = 36)
Length—range (cm)	4.8–12.5	1.5–5.5
Length—mean (cm)	6.9	2.9
Diameter at origin—range (cm)	0.8–1.3	0.7–1.0
Diameter at origin—mean (cm)	0.97	0.82
Diameter at termination—range (cm)	1.0–1.8	0.8–1.5
Diameter at termination—mean (cm)	1.22	1.05
Cases analyzed (*n*)	42	36

**Table 8:** Types of venous anastomosis among the 77 kidneys with at least one communicating channel (both sides combined).

Type of Anastomosis	*n*/*N* (%)
Extrarenal, intertruncular	31/77 (40.3)
Juxtahilar, anterior	19/77 (24.7)
Intrasinusal (intrarenal)	14/77 (18.2)
Retroaortic, curved path	8/77 (10.4)
Other (lobar or segmental)	5/77 (6.5)

Each kidney was classified by its principal type of communication.

**Table 9:** Summary of clinically relevant renal vein anatomical variants identified.

Variant	*n*/*N*	% (95% CI)	Side	Anatomical Note
Multiple renal veins (≥2 caval ostia)	16/116	13.8 (8.7–21.2)	Right 14/52 (26.9); left 2/64 (3.1)	Markedly more common on the right side
Retroaortic left renal vein	2/42	4.8 (1.3–15.8)	Left	Anatomical variant, not nutcracker syndrome
Complete circumaortic venous ring	1	Not estimable	Left	Single descriptive case; denominator unavailable

## Data Availability

The original contributions presented in this study are included in the article. Further information on the retained records is available from the corresponding authors upon reasonable request. Individual-level morphometric records and duplicate measurement readings were not retained and cannot be provided.
